# Reflections on a range of cardiovascular issues

**Published:** 2013-11

**Authors:** Paul A Brink

**Affiliations:** Department of Internal Medicine, Faculty of Health Sciences, University of Stellenbosch and Tygerberg Hospital, Tygerberg

Articles in this issue range from epidemiology and determinants of risk factors for non-communicable diseases, namely, the prevalence of hypertension in Nigeria (Murthy *et al.*, page 344), to obesity in South African woman (Micklesfield *et al.*, page 369) and risk determination of cardiovascular events in persons with diabetes mellitus (Kengne, page 376). Interesting cardiac perfusion abnormalities are addressed in three articles (Tasolar *et al.*, Celik *et al.* and Chang *et al.*, pages 355, 357, e12), which are complimented by an article on congenital coronary anomalies (Karabay *et al.*, page 351) as detected by CT-scanning. Furthermore, two ways of creating a shunt for renal dialysis purposes using the basilica vein is discussed by Ozcan *et al.*. (page 364).

Murthy *et al.* in a well-designed survey of blindness and visual impairment in Nigeria, piggy-backed hypertension onto the study. They found a prevalence of hypertension of 44.9% in persons over 40 years, which varied across ‘ethnic’ groups, the Kanuri group having the highest prevalence of 77.5%.

This begs the question of ethnicity, culture and race, the latter being historically of significant concern in South Africa, and brought to the fore by Micklesfield *et al.* in a review on factors determining obesity in black South African women. These concepts overlap, however, and there do not appear to be clear definitions. The South Africa government classifies four racial groups, namely, black, white, coloured (mixed ancestry) and Indian.

Nevertheless, black African women in South Africa have an obesity prevalence of 31.8%, which is the highest in sub-Saharan Africa. Micklesfield and co-workers discuss the role of various factors, such as socio-cultural, behavioural, maternal and early life, socio-economic status, education, physical activity (or lack thereof), gender, urbanisation, eating behaviour and body image. Genetics, so fashionable currently, certainly does not appear to be a major determinant of obesity.

Kengne grapples with risk prediction in diabetes mellitus (DM); risk prediction that will be valid globally. He describes the ADVANCE (Action in Diabetes and Vascular disease: PreterAx and DiamicroN MR Controlled Evaluation) study, with which he was intimately involved. Diabetes mellitus is not just another risk factor to be added to other cardiovascular risk factors for cardiovascular disease (CVD), but is, rather, a condition with a gradient of influences. Therefore, a need for DM-specific risk determination is necessary.

The ADVANCE study was a multi-centre study of persons with DM and therefore CVD risk, which led to a prediction model that has been both internally and externally validated. Formulae, such as those derived from the Framingham project, tend to overestimate risk in persons with DM. A calculator for calculating CVD risk in DM is available online (http://www.advanceriskengine.com/).

Two groups from Turkey (Tasolar *et al.* and Celik *et al.*) address the phenomenon of slow coronary flow, an angiographic finding characterised by delayed distal vessel opacification in the absence of significant epicardial coronary disease. One article is about the role of endothelial nitric oxide synthase levels and the response to exercise, while the other is on the role of vitamin E and antioxidant activity in this phenomenon.

The case study of Chang *et al.*, published online, is about Kounis syndrome, defined by either cardiogenic shock or an acute coronary syndrome due to vaso-activity triggered by the release of inflammatory mediators following an allergic insult.

So, overall, this edition of the journal has an interesting mix of articles with which to conclude 2013. Best wishes for the year to come.

